# 

**Published:** 1909-05-29

**Authors:** 


### Bequests and Donations.

The Goldsmiths' Company has sent £50 to the British Lying-in Hospital, Endell Street, W.C., which is in great need of assistance, to reduce the debt due to the bankers.

The Treasurers of the Middlesex Hospital have received from the Salters' Company the half-yearly instalment of £50 for the maintenance of the scholarship attached to the cancer research laboratories of the hospital.

Lord Sandhurst, Treasurer of St. Bartholomew's Hospital, has received from Mr. H. J. Waring, F.R.C.S., Senior Assistant-Surgeon, a donation of fifty guineas towards the new pathological block.

Dr. James Hurd Keeling, M.D., F.R.C.S., of Sheffield, who died last March, aged 77, left estate of the gross value of £11,480, and bequeathed £250 each to the Jessop Hospital for Women, Sheffield, and the Sheffield University.

Mr. John Smith, of Monifieth, Forfarshire, merchant, left £2,000 to the Dundee Royal Infirmary, £1,000 to the Dundee Royal Victoria Hospital for Incurables, and £500 to the Gerard Cottage Hospital, Monifieth.

The late Mr. Frederick Gorringe, chairman of Messrs. F. Gorringe, Ltd., left £5,000 to the Bolingbroke Hospital, Wandsworth Common, and the residue of his estate, which will amount to about £400,000, is to be divided equally among the Westminster Hospital, St. George's Hospital, and six other charitable institutions.

Alderman William Robinson, of Salford, Lancashire, who died on March 29, aged seventy-one, left remainder of £10,000, on the decease or remarriage of his niece, of £5,000, to the Salford Royal Hospital, Salford; £2,000 to the General Hospital and Dispensary for Sick Children, Pendlebury; and a sum, estimated at £2,000, to the Salford Royal Hospital, Salford.

Mr. Edwin Gayford, of Piccadilly, has bequeathed £1,000 to the Hospital Sunday Fund.

### THE GRADUATES' MEDICAL SCHOOLS.

DIARY FOR THE WEEK, MAY 31 TO JUNE 5.

**ROYAL SOCIETY OF MEDICINE, 25 Hanover Square, W.**

**At 5 p.m.**
**June 4,** Laryngological Section, **Cases and Specimens.**

**NORTH-EAST LONDON POST-GRADUATE COLLEGE, Prince of Wales's General Hospital, Tottenham, N.**

**At 4.30 p.m.**
**June 1,** Dr. G. P. Chappell, **Demonstration of Selected Medical Cases.**

**THE THROAT HOSPITAL, Golden Square, W.**

**At 5.30 p.m.**
**June 3,** Mr. Faulder, **Surgical Anatomy of the Tympanum, Labyrinth, and Mastoid.**

**NATIONAL HOSPITAL FOR THE PARALYSED AND EPILEPTIC, Queen Sq., Bloomsbury, W.C.**

**At 3.30 p.m.**
**June 4,** Mr. Sargent, **Decompressive Operations.**

**LONDON SCHOOL OF CLINICAL MEDICINE, Seamen's Hospital, Greenwich, S.E.**

**At 3.15 p.m.**
**June 1,** Mr. Carless, **Fractures in the Region of the Wrist, and their Treatment.**
**At 2.15 p.m.**
**June 2,** Dr. F. Taylor, **Myelitis.**
**At 2.30 p.m.**
**June 3,** Dr. Rankin, **The Neurotic Element in Disease.**

**THE POST-GRADUATE COLLEGE, West London Hospital, Hammersmith, W.**

**At 10 a.m.**
**June 3,** Surgical Registrar, **Demonstration.**
**June 4,** Medical Registrar, **Demonstration.**
**At 12.15 p.m.**
**June 1,** Dr. Pritchard, **Practical Medicine.**
**June 2 and 5,** Dr. Grainger Stewart, **Practical Medicine.**
**At 5 p.m.**
**June 1,** Dr. Saunders, **Clinical Lecture, with Cases.**
**June 2,** Dr. Beddard, **Medicine.**
**June 3,** Dr. Cole, **Acute Mania and its Treatment.**
**June 4,** Dr. Pritchard, **Infective Endocarditis.**

**MEDICAL GRADUATES' COLLEGE AND POLYCLINIC, 22 Chenies Street, W.C.**

**At 4 p.m.**
**June 1,** Dr. C. O. Hawthorne, **Medical.**
**June 2,** Mr. Edred Corner, **Surgical.**
**June 3,** Sir Jonathan Hutchinson, **Surgical.**
**June 4,** Mr. Harold Barwell, **Ear, Nose, and Throat.**
**At 5.15 p.m.**
**June 1,** Mr. Herbert Eason, **The Pupil in Disease.**
**June 2,** Dr. Charles Mercier, **The Diagnosis of General Paralysis in its Early Stage.**
**June 3,** Dr. Essex Wynter, **Treatment of Chorea.**

THE HOSPITAL May 29, 1909.

*Name* ..............................

*Address* ..............................

..............................

This Coupon must accompany manuscript or contributions intended for The Hospital.

